# A Single-Center Sham and Active-Controlled Double-Blind Randomized Crossover Trial of the Magnetic Levator Prosthesis for Severe Blepharoptosis

**DOI:** 10.1167/tvst.14.2.15

**Published:** 2025-02-11

**Authors:** Kevin E. Houston, Shrinivas Pundlik, Prerana Shivshanker, Alex R. Bowers, Sarah LaRosa, Mara Robinson, James Chodosh, Lynn Brandes, Patrick Lee, Eleftherios I. Paschalis

**Affiliations:** 1 Department of Veteran Affairs, Central Western Massachusetts, Optometry Service, Worcester, MA, USA; 2 Departments of Neurology and Ophthalmology, University of Massachusetts Chan Medical School, Worcester, MA, USA; 3 Harvard Medical School Department of Ophthalmology, Schepens Eye Research Institute of Massachusetts Eye and Ear, Boston, MA, USA; 4 Brooks Rehabilitation Center for Low Vision, Jacksonville, FL, USA; 5 Massachusetts Eye and Ear, Facial Nerve Clinic, Harvard Medical School Department of Otolaryngology, Boston, MA, USA; 6 Department of Ophthalmology and Visual Sciences, University of New Mexico, Albuquerque, NM, USA; 7 Massachusetts Eye and Ear, Vision Rehabilitation Clinic, Harvard Medical School Department of Ophthalmology, Boston, MA, USA; 8 Massachusetts Eye and Ear, Department of Ophthalmology, Boston Keratoprosthesis Laboratory, Boston, MA, USA

**Keywords:** blepharoptosis, ptosis, eyelid, magnet, device

## Abstract

**Purpose:**

To evaluate the safety and efficacy of the magnetic levator prosthesis (MLP) relative to active control with KT Tape, an elastic therapeutic tape used clinically to mechanically open the lids, and to a sham MLP worn in-office only.

**Methods:**

This was a double-masked, randomized crossover single-center trial of patients with severe unilateral or bilateral paralytic blepharoptosis defined as occlusion of the visual axis without frontalis recruitment. Patients were allocated to MLP or tape first and then crossed over after 2 weeks of use and a 2-week washout. Primary outcome was maximum eyelid closure on spontaneous blink measured in video frames with ImageJ. Primary patient-reported outcome was the Glasgow Benefit Inventory and, secondarily, comparison of the amount of eye opening and proportions of complete volitional blinks.

**Results:**

Of 16 patients randomized, 15 completed the crossover. MLP and tape equally improved eye opening over sham (MLP, 6.8 mm [95% confidence interval (CI), 5.2–8.4]; tape, 7.0 mm [5.4–8.6]; sham, 3.9 mm [2.3–5.5], all *P* < 0.001). Spontaneous blinks were significantly better with MLP (2.4 mm [95% CI, 1.5–3.7] compared to tape, 4.1 mm [2.6–6.5], *P* < 0.001). Incomplete volitional blinks were much more common when wearing tape compared to when wearing the MLP (*P* < 0.001), which was not different from sham. There was a significant perceived benefit of both MLP and tape (*P* < 0.001). There were three related nonserious adverse events over 3539 hours of tape use and two related nonserious adverse events over 4632 hours of MLP use.

**Conclusions:**

The MLP was superior to KT tape and sham for the treatment of severe blepharoptosis.

**Translational Relevance:**

First randomized controlled clinical trial of the MLP. (https://clinicaltrials.gov/study/NCT04678115?cond=Blepharoptosis&term=kinesiotape&rank=1, NCT04678115)

## Introduction

Severe blepharoptosis, defined as incomplete opening of the upper eyelid, which blocks the visual axis without frontalis recruitment,^1^ occurs due to abnormalities in the levator palpebrae superioris muscle, dysfunction of the superior division of the third cranial nerve, or structural abnormalities.^2^ Etiologies include but are not limited to congenital abnormalities, traumatic brain injury, tumors of the brain or periocular region, stroke, viral illnesses, demyelinating diseases, diabetes, autoimmune disorders such as myasthenia gravis, and mechanical causes, including those due to aging.^2^^,^^3^ The prevalence of severe blepharoptosis is unknown; however, in Korean and UK general populations, blepharoptosis of any severity has been reported at 11%.^4^^,^^5^ If only 1% of all blepharoptosis cases are severe to total, then 330,000 in the United States and 9 million globally are affected.

The most common method currently used to correct ptosis involves surgical levator advancement or, in more severe cases, frontalis sling.^2^^,^^3^ While these procedures are well accepted, there is a trade-off between the amount of eyelid elevation and risk of lagophthalmos, which can result in chronic exposure keratoconjunctivitis and related complications. As a result, a conservative surgical approach is typically employed by leaving the blepharoptosis undercorrected, particularly in total levator palsy. Patients with severe ptosis are in dire need of better treatment options, and a nonsurgical approach is desirable, as full eye opening can be provided and then the intervention could be easily disengaged or adjusted when needed to maintain ocular surface health. Currently available temporary or nonsurgical treatments include taping the lid(s) and propping the eyelid open with a wire on the glasses (ptosis crutch), neither of which restores normal blink function.^6^^,^^7^

To improve upon available treatment options, we developed the magnetic levator prosthesis (MLP).^1^^,^^7^^–^^10^ The force to lift the lid is produced by a static neodymium (NdFeB) magnet embedded in a glasses frame and an array of two to three small magnets embedded in polydimethylsiloxane elastomer (PDMS) fitted externally to the upper eyelid with intravenous (IV) securement film. The film is approved by the US Food and Drug Administration (FDA) for extended wear on the skin and as an eye covering.^11^ In prior studies, it generated a strong bond, keeping the magnetic array affixed to the eyelid skin for a mean of 6 ± 4 days with good patient-reported comfort when used for 2 hours per day during rehabilitation therapies.^7^ We later introduced an adjustable magnetic force design comprising a simple manual dial on the side of the spectacle frame, which was effective to customize the attractive force magnitude and direction without having to exchange magnets.^9^^,^^10^

The next step in this project, reported here, was to conduct a double-blind sham and active-controlled clinical trial. This included a crossover comparison of the MLP to the most common clinically used nonsurgical and temporary approach, elastic therapeutic tape, well-known by the trade name KT Tape (American Fork, UT, USA), or generically as kinesiotape. KT Tape is widely used by physical therapists and sports medicine specialists,^12^ as well as in rehabilitation facilities for facial palsy and severe blepharoptosis, primarily by occupational, physical, and speech therapists. In this study, KT taping was compared to the MLP and a sham MLP with the primary hypothesis that the MLP and tape would provide better eye opening than the sham but that the MLP would allow better eye closing during blinking. A patient-reported outcome, the Glasgow Benefit Inventory (GBI), was also compared between the interventions.

## Methods

### Trial Design

**Figure 1.**
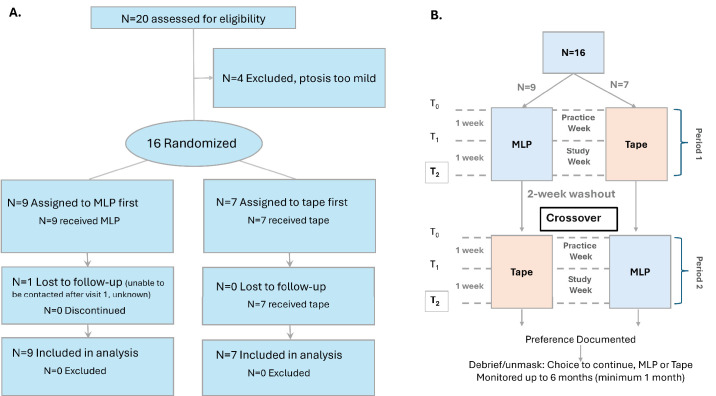
(**A**) CONSORT study diagram. (**B**) Detailed study diagram illustrating crossover and time points. Primary analyses conducted for measures taken at T_2_. Safety monitored throughout the study.

Data for this study were collected at Massachusetts Eye and Ear between 2020 and 2023. A phase II equivalent double-blind, single-center, randomized controlled crossover clinical trial was conducted to determine the safety and efficacy of the MLP compared to KT Tape frontalis sling to open the ptotic eyelid while allowing blinking. An AB/BA sequence was used with outcome measurements collected during in-clinic visits at baseline with no intervention, immediately with the MLP or tape (T_0_), after a practice week (T_1_), and after another week (study week, T_2_) (Fig. 1B). A table of outcomes is available in Supplement 1. A sham MLP was also tested at MLP T_1_ to account for any placebo effect. The sham was an MLP frame that looked identical and had similar weight but had no magnetic properties and a real eyelid array device. A washout period of 2 weeks was used to eliminate any skin erythema or ocular surface irritation that may have occurred with the device worn in the first period of the crossover.

#### Extended Device Use and Monitoring

Participants were allowed to continue for up to 6 months with either the KT Tape or the MLP (their preference), so long as the chosen intervention was not graded as unsafe by a study clinician or an independent clinician safety monitor. They returned approximately monthly where safety and efficacy were monitored. Decisions to stay in the study and continue to be monitored were primarily based on transportation and time availability factors of the participants; however, the study clinician and monitor could advise against continuing if there were safety or efficacy concerns. Potential longer-term effects of the interventions were evaluated through phone calls at 6 and 12 months for all cases, including those that did not select extended device use.

### Participants

The study population had severe unilateral or bilateral ptosis defined as occlusion of the visual axis by the lid in the resting state without frontalis recruitment and ability to provide informed consent or assent. A typical interpalpebral fissure height (IPF, distance between upper and lower lids at the visual axis in primary gaze) in the unaffected eye of patients with severe ptosis is 10 to 12 mm.^1^^,^^7^^,^^9^^,^^10^ Those with severe ptosis typically have an IPF in the affected eye(s) of 6 mm or less. Characteristics are summarized in Table 1.

**Table 1.** Participant Characteristics

Characteristic	Value
Number of participants	15^a^
Age, y	
Median	61
25th to 75th percentile	55–65
Minimum/maximum	13/80
Gender, female, *n* (%)	6 (40)
Race, White, *n*	15
Ptosis etiology, *n*	
Unilateral third nerve palsy	5
Myasthenia gravis	4
Bilateral third nerve palsy	2
Congenital ptosis, oculopharyngeal muscular dystrophy, craniosynostosis, and chronic progressive external ophthalmoplegia	1 each^b^
Baseline (sham) resting state opening IPF, mean (SD), mm	3.89 (2.36)

a Total enrollment: 16, 1 lost to follow-up after the first visit in the first crossover arm; primary outcome included data for 15 participants.

b Three acute cases were enrolled.

### Interventions

#### Device Description and Classification

**Figure 2.**
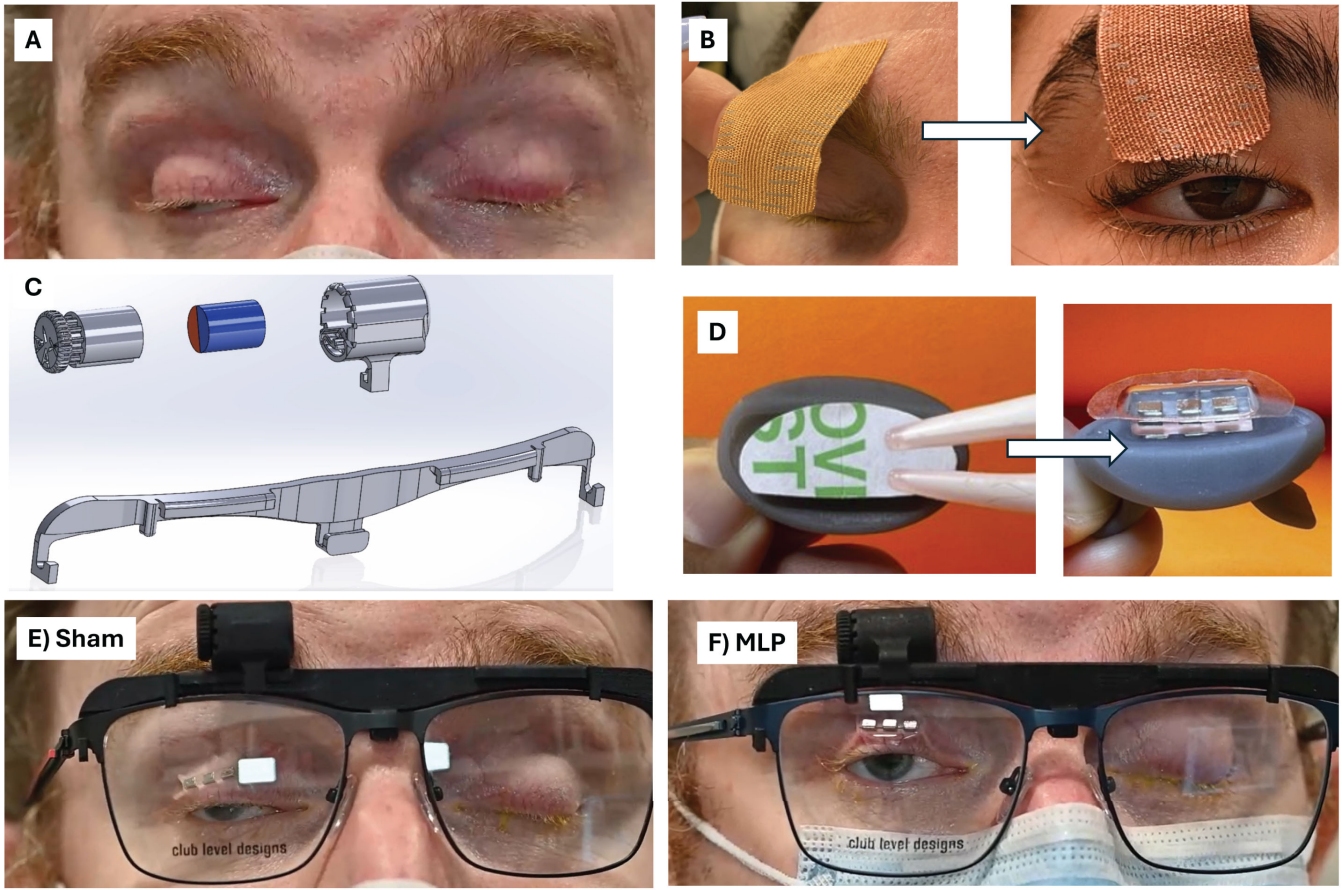
(**A**) Participant with profound bilateral blepharoptosis at baseline. (**B**) KT Tape application procedure. Tape is anchored on the frontalis area and stretched to approximately 50% tension to just above the eyelid margin. (**C**) Exploded schematic of the MLP clip showing adjustable dial and diametrically polarized 9.5-mm × 12.7-mm N52 cylinder where *red* was north and *blue* was south. (**D**) Eyelid array device and application procedure. The array consisted of two or three 3-mm × 2-mm × 1-mm N52 magnets encapsulated in PDMS elastomer (Sylgard 184; Dow Corning, Midland, MI, USA) and bonded to transparent medical adhesive (Opsite Flexifix; Smith & Nephew, London, UK) cut to the size and shape of the upper eyelid using a cutting plotter machine. For application, the white backing was removed with plastic tweezers to expose the adhesive side of the clear Opsite Flexifit film. This was applied to the eyelid with the gray applicator shown. Tweezers could also be used to remove the device. (**E**) The participant in (**A**) with sham MLP. (**F**) The same participant with the active MLP, which permitted clearing of the visual axis while maintaining a full and natural blink.

The MLP is a noninvasive and easily removable device consisting of neodymium magnets (SM Magnetics, Pelham, AL, USA) 52 MGOe (1.44 T) embedded in bioinert PDMS. The MLP is a class 1 device, not requiring an investigational device exemption for use in the study. KT Tape is commercially available without prescription and is registered as class 1 for use on the skin to support sore or weakened muscles. Three consultants with KT Taping expertise were involved in fitting the KT Tape and used methods advised by the International Kinesiotaping Association,^12^^–^^14^ providing appropriate skill while minimizing bias. Devices are shown in detail in Figure 2, with additional specifications and fitting techniques provided in Supplement 2.

#### Fitting and Application of the MLP

The eyelid was prepared with OCuSOFT lid scrub (OCuSOFT, Rosenberg, TX, USA).

Self-application training: A participant training session was performed for both devices described in detail in Supplement 3.

### Outcomes

See Supplement 1 for a table of outcome measures.

#### Primary Outcome

##### Spontaneous Blink Maximum Closure

The primary outcome was objective measurement of the IPF midpoint height during maximum closing in five spontaneous blinks, captured with a Nikon mirrorless SLR video recording system (Z7II; Nikon USA, Melville, NY, USA), with a visible light source mounted just above the lens, measured manually with ImageJ (National Institutes of Health, Bethesda, MD, USA). The IPF in severe ptosis will typically be less than 6 mm (Table 1). Spontaneous blinks are normally incomplete, a behavior that is dependent upon age, gender, task, and environmental demands.^15^ Maximum closing was defined as the three points at the base of the blink, as described in detail in a prior study.^10^ Participants were instructed to look at the camera lens and *try to relax and behave normally*. Recording was stopped after 1 minute or after five spontaneous blinks, whichever came first. Recording always occurred in the same windowless clinical research room with consistent lighting, airflow, and humidity level.

#### Secondary Outcomes

##### Volitional Blink Maximum Closure

At the end of recording, participants were instructed to close their eyes completely and open completely three times, representing volitional blink trials. These were measured manually with ImageJ.

##### Resting Open

Resting open IPF was the median between blink-event IPF, measured automatically by a deep learning model for eyelid landmark detection.^16^

##### Glasgow Benefit Inventory 5f

Relative patient-reported benefits and harm of the interventions were evaluated using the revised 15-question five-level GBI. Scores can range between –100 and 100, with 0 indicating no benefit, negative values indicating worse, and positive values indicating higher benefit.^17^

##### Wear Time

Wear time was documented using a daily log. Additionally, participants estimated their daily average wear time at each study visit. Total hours and days of wear were computed for each participant. During the crossover phase, a text message survey was sent twice daily at random times in the morning and afternoon asking if the device was being worn. Apparent discrepancies were flagged and reported.

#### Safety Outcomes

Systemic and ocular safety were monitored at each encounter for adverse events. Reporting criteria were skin erythema >3/7,^18^ corneal staining worsening >1.5 points^19^ or any ulceration, conjunctival staining worsening >2 points, visual acuity decrease >2 lines, or comfort rating <5/10. Skin erythema was measured using an FDA industry scale (0–7). Visual acuity^20^ was measured with spectacles and pinhole. Comfort was monitored with a Likert-type rating scale (1 = worst to 10 = best),^7^ with comfort <5 being a safety cutoff. All slit-lamp assessments were performed with sodium fluorescein and video recorded with an iON system (Marco, Jacksonville, FL, USA), and all safety events were reviewed by one of the independent safety monitors.

### Sample Size

Sample size was estimated based on the treatment condition (three-level factor) effect size on the spontaneous blink IPF measurements (0.55) after eight participants had completed the crossover using one-way analysis of variance power calculation. This led to a sample size of 12 participants with power of 0.80 and type II error of alpha 0.05. To account for possible attrition, 16 were enrolled, with 15 completing the crossover.

### Random Sequence Generation and Allocation

Participants were assigned to one of two possible treatment allocations using MinimPy software (Python Software Foundation, Fredericksburg, VA, USA) that implemented the process of minimization: (1) MLP and then KT Tape or (2) KT Tape and then MLP. The first participant was assigned randomly with subsequent participants order-balanced for age ±65 years and acute versus chronic blepharoptosis.

### Masking

Participants did not know which device was experimental versus active control or sham, and data analysts were masked to treatment groups (see Supplement 4 for details).

### Statistical Methods

Linear mixed-effects regression was used to determine the effect of treatment condition on the spontaneous blink and resting state open IPF, with participant as a random intercept. Covariates of age, gender, blink sequence (order of the blink), and the order of interventions (first or second arm of the crossover) were investigated alone, and significant (*P* < 0.05) covariates were included in the final model, from which the estimated marginal means and their 95% confidence interval were reported for each of the outcomes. In a post hoc analysis, the association of treatment condition with spontaneous blink IPF was controlled for the baseline (sham) eyelid-opening IPF at 1 mm and 6 mm, representing milder and more severe ptosis. For volitional blinks, a test of proportionality was performed to determine the effect of the three treatments on the probability of the eyelid not fully closing. Paired *t*-test was used to determine the difference in the mean GBI scores between MLP and tape.

The full trial protocol is available in Supplement 5 and includes a study grid showing procedures performed at each visit.

## Results

### Blink Results

**Figure 3.**
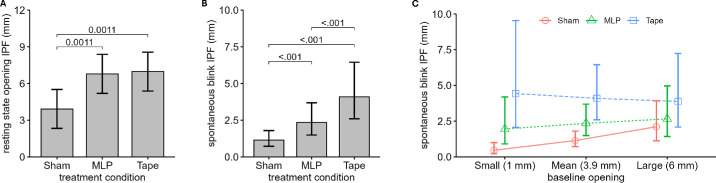
IPF postestimation data plots (mean and 95% confidence interval) comparing the different treatment conditions. (**A**) Both MLP and tape provide significant opening compared to sham. (**B**) Spontaneous-blink IPF at mean baseline opening value (of the sample) showing that MLP allows better (smaller) IPF but still less closure than the sham control. (**C**) Spontaneous-blink IPF across the three conditions over the range of baseline eye opening, indicating that MLP permitted significantly better closure than tape at all severity levels of blepharoptosis.

All IPF results with *P* values and estimates are shown in Table 2 and Figure 3. To summarize, both tape and MLP significantly improved eyelid opening compared to sham, but there was no difference between MLP and tape. For spontaneous blinks, MLP allowed better closing than tape, regardless of baseline severity of ptosis. For volitional blinks, there was significantly better closing with MLP compared to tape, with 16 incomplete closures with tape compared to 3 with MLP and 1 with sham (all out of 45). Participant age, gender, and blink sequence were not statistically significant predictors for any of the outcomes.

**Table 2.** Results Summarizing the Comparison of Observed and Patient-Reported Outcomes Between the Three Conditions

		Estimates			*P* Values for Comparisons		
Outcome	Type of Quantity	MLP	Tape	Sham	MLP vs. Tape	MLP vs. Sham	Tape vs. Sham
s-blink IPF	EMM (95% CI) at mean baseline = 3.96 mm	2.4 (1.5–3.7)	4.1 (2.6–6.5)	1.1 (.7–1.8)	<0.001	<0.001	<0.001
	EMM (95% CI), baseline = 1 mm indicating higher severity of ptosis	1.9 (.9–4.2)	4.4 (3.1–9.5)	0.5 (.2–1.0)	<0.001	<0.001	<0.001
	EMM (95% CI) baseline = 6 mm indicating less severe ptosis	2.7 (1.4–5.0)	3.9 (2.1–7.2)	2.1 (1.3–3.9)	0.016	0.09	<0.001
open IPF	EMM (95% CI)	6.8 (5.2–8.4)	7.0 (5.4–8.6)	3.9 (2.3–5.5)	0.81	0.001	0.001
v-blink nonclosure	Proportion	0.067	0.356	0.022	0.61	0.004	<0.001
Comfort	EMM (95% CI)	8.5 (7.2–9.7)	8.0 (6.7–9.2)	9.2 (7.9–10.4)	0.37	0.31	0.11
GBI^a^	Mean (95% CI)	21.8 (14.4–29.2)	14.4 (7.0–21.9)	—	0.097	—	—

CI, confidence interval; EMM, estimated marginal mean; open IPF, resting state opening; s-blink, spontaneous blink;

v-blink, volitional blink.

a GBI scores were obtained only for MLP and tape and not for sham.

### Wear Time, Crossover Phase

Mean (SD) wear time with tape was 20.6 (11.1), days with a mean (SD) total of 136.3 (102.0) hours of wear, amounting to an average of 6.3 (2.3) hours of wear per day. MLP wear time was 28.1 (16.7) days and mean total of 154.1 (97.7) hours, with an average of 5.7 (2.4) hours of wear per day. The rate of wear was not significantly different between MLP and tape (*P* = 0.38). Text messaging wear time survey supported accurate reporting for all but two participants, one during the tape phase and one during MLP phase.

### Safety Outcomes, Crossover Phase

There were no safety events during the crossover phase for the MLP or sham. There were three related nonserious adverse events for tape, including an increase in skin erythema scale greater than 3 out of 7 for two participants and one increase in conjunctival staining >2 points. All resolved with conservative management. There was one case of ocular migraine during washout that was unlikely related.

### Patient-Reported Outcomes

There were no significant differences in comfort rating between conditions (Table 2) (Supplement 6). Both MLP and tape were perceived as beneficial (GBI significantly >0), with MLP marginally better than tape (*P* = 0.097, Table 2). There was no difference in device preference (seven preferred MLP, six preferred tape, and two preferred neither). The reasons for preferring MLP were it helped to open the eye(s) more (*n* = 3), provided better comfort (*n* = 2), was more cost effective (*n* = 1), and was cosmetically better (*n* = 1). The reasons for preferring tape were cosmetically better/did not have to wear glasses (*n* = 3), works better (*n* = 2), and less weight/better comfort (*n* = 1).

### Extended Use Outcomes

Eight participants (50%) participated in extended use, three with tape and five with MLP. Seven declined citing time commitment (*n* = 4), personal decision (*n* = 2), and loss to follow-up (*n* = 1). Wear time during the extended phase was not different (*P* > 0.03), with tape mean (SD) wear time of 670.7 (323.1) hours over 112.7 (51.5) days and MLP mean (SD) 464.2 (206.1) hours over 79.0 (30.9) days. There were no serious or systemic related or unrelated events, and there were two related nonserious events for MLP, including one skin erythema rating >3/7 and one increase in corneal staining >1.5 points.

### Safety Outcomes: Total Adverse Events

In total, including crossover and extended use phases, for tape, there were three related nonserious adverse events over 3539 hours of use, and for MLP, there were two related nonserious adverse events over 4632 hours of use.

## Discussion

This study provides evidence to support the MLP as a superior treatment to KT Tape for severe to total unilateral or bilateral paralytic blepharoptosis. Both interventions improved eyelid opening compared to sham, but the MLP allowed better eye closure during both spontaneous and volitional blinks, which should lead to a healthier and more comfortable intervention in the long run. Despite better blinking with the MLP, in the limited time frame of this study, there was not a clear difference in adverse events between interventions. Further research with monitoring of the longer time of use could be helpful to determine the relative benefits of a better blink; however, there is already a large body of literature supporting the importance of high-quality blinking on long-term health and function of the ocular surface.^21^ It was considered that that tape might be superior to MLP for milder cases of ptosis, but this was not supported by the results (Table 2, shaded row), which found the MLP allowed better spontaneous blink at all levels of severity.

The feasibility of both the MLP and KT Tape was supported by this study. Participants were able to self-apply the devices after one short in-office training session, and none discontinued because of difficulty with application. Additionally, there were no device durability issues or loss of efficacy over time. Careful documentation and verification of device usage with twice-daily text messaging surveys during the crossover was a strength of this study, strongly suggesting the interventions were used on a regular basis. We considered utilizing a sensor to objectively measure usage; however, it would have only been possible with the MLP and would have increased the weight. At this time, the best possible approach is to use random text messaging surveys, as was done here. To our knowledge, this is the first use of such an approach in a vision rehabilitation device study. Text messaging could be used in similar studies going forward.

The safety of both devices was excellent, with only three and two related nonserious adverse events for tape and MLP, respectively. All events were able to be managed by improving the fit, reducing wear time, and/or adding ocular lubricants, and no participants had to be removed from the study. Skin erythema, when it occurred, was related to mechanical forces rather than as a hypersensitivity response, which has not been an issue in this and prior studies of blepharoptosis.^1^^,^^7^^,^^8^^,^^10^

While the MLP was objectively much more effective than the tape for blinking, patient-reported outcomes did not find clear superiority in this relatively short period of use. The GBI scores were only marginal, suggesting there may be a small perceived benefit for the MLP; however, a larger study is needed to further evaluate this trend. Almost half of participants actually preferred the KT Tape, mostly because they did not want to wear glasses or did not like the weight of the frame. A spectacle option with better balancing of weight was found to be very feasible using custom three-dimensional printed MLP frames^8^ and could be utilized in future studies to address this complaint. Furthermore, the superior blink function made possible by the MLP is likely to provide better eye health and vision in studies with longer usage times.

### Limitations and Future Directions

Sample size was limited in diversity, and only five clinicians were involved in fitting and training participants with the devices. A larger, multicenter version of this study with longer usage times is warranted given the positive results and would be required to determine applicability of the findings to clinical practice.

## Conclusions

The MLP was superior to KT Tape and sham for the treatment of severe blepharoptosis in this sample.

## Supplementary Material

Supplement 1

Supplement 2
