# Supplementary material for: A Single-Center Sham and Active-Controlled Double-Blind Randomized Crossover Trial of the Magnetic Levator Prosthesis for Severe Blepharoptosis
Source: Transl Vis Sci Technol. 2025 Feb 11;14(2):15. doi: 10.1167/tvst.14.2.15 (PMC11817847; doi:10.1167/tvst.14.2.15)
Supplement: Supplement 1 [file tvst-14-2-15_s001.docx]

**Supplement 1: Outcome Measures Table**

| **Table S1: Outcome Measures** | | | |
| --- | --- | --- | --- |
| **Outcome Name** | **Type** | **Number of Data Points per condition** | **Timepoints for Analysis** |
| **Primary Outcome** | | | |
| Maximum Closure, Spontaneous Blink IPF | Objective, quantitative, continuous | 5 | T2 |
| **Secondary Outcomes** | | | |
| Maximum Closure, Volitional Blink IPF | Objective, quantitative, continuous | 3 | T2 |
| Resting Open IPF | Objective, quantitative, continuous | hundreds | T2 |
| Related Adverse Events | Counts | Over thousands of hours of use | All |
| Glasgow Benefit Inventory | Patient reported outcome measure, ordinal | 1 | T2 |
| Device Usage Monitoring | Text message survey, random sampling, Binary | 14 | All |

**Supplement 2: Detailed Description of Devices**

MLP Device

All magnet components were produced with NdFeB 52 MGOe (1.44 T).

The eyelid component of the device was comprised of arrays of 2 or 3 rectangular cubes (3 mm × 2 mm × 1 mm Length x Width x Height), encapsulated in 10:1 ratio PDMS elastomer (Sylgard 184; Dow Corning, Midland, MI, USA) cured per manufacturer instructions at 65-80° Fahrenheit in a laboratory grade oven. Arrays were produced in a mold yielding batches of 30 arrays which were hand cut to rectangular cubes of approximately 13 x 4 x 3mm (Length x Width x Height). The cut arrays were covalently bonded to the outside non-adhesive side of transparent medical adhesive film (Opsite Flexifix; Smith & Nephew, London, UK) by dipping them in 10:1 PDMS, placing on a swatch of the film cut to the size and shape of the upper eyelid using a cutting plotter machine (Cricut, South Jordan, UT, USA), and cured at 65-80° Fahrenheit. The Opsite Flexifit is FDA approved for extended wear on the skin by predicate Tegaderm labeling (3M corporation, St. Paul, MN, USA).

The spectacle magnet was cylindrical (9.5 mm × 12.7 mm) and magnetized through the diameter (SM Magnetics, Pelham AL, USA). It was inserted in a three-dimensional (3-D) printed enclosure on the spectacle frame (3D Hubs, Amsterdam, Netherlands), shown in manuscript Figure 2. The spectacle magnet could be manually rotated with the fingers, allowing adjustment of the magnetic force between the eyelid and spectacle magnets.

Fitting and Application of the MLP: The eyelid was prepared with Ocusoft lid scrub (OcuSoft, Rosenberg, TX) and the eyelid component applied as shown in Figure 2D of the Manuscript. The glasses were donned, the spectacle magnet positioned directly above the array (Figure 2C), and the force dial adjusted to achieve the best opening without impeding the blink.

KT Taping Description and Classification

KT Tape is an elastic skin tape FDA registered as a 510K exempt class 1 device, typically used by physical and occupational therapists to support muscular healing and movement. It is well known for use in sports injuries and is sometimes used on the face in cases of facial palsy or on the eyelid to support the lid in cases of severe blepharoptosis. In such cases it may be attached near the lid margin above the lashes extending up to the forehead skin overlying the frontalis muscle. The Tape is fairly easily removed. Other brands of kinesiotherapy tape are available; however, in this study the KT Tape brand was utilized (KT Tape, American Fork, UT).

Fitting and Application of the KT Tape: The use of therapeutic elastic tape for the facilitation of eyelid opening is not a forced eyelid approach. The Tape was applied in such a way to theoretically provide afferent feedback via the integumentary and nervous system to produce an efferent motor response, as describe in prior KT Taping educational materials(14). The tape is used as a reminder to the patient to try to actively recruit the levator muscle throughout the day in trying to open their eyelid. As such, manufacturer recommendations were followed by stretching with low-moderate (25-35%) tension through the body of the tape when applying to the eyelid skin(14). Tape was provided for fitting in 6 sizes, the dimensions of which were advised by the KT Taping consultants, (Width x Height(cm)): 1.5x4, 1.5x5, 2x3, 2x4, 2x5, 2.5x5). Study staff were trained and supervised by one of the KT Taping consultants. The initial anchor adhered cranially was applied to the forehead skin just above the eyebrow without tension, then low-moderate tension (25-35%) on tape without a release of any stretch was applied throughout the body of the tape prior to the caudal anchor being adhered just above the lash line without any tension on tape. This placed a “recoil” tension toward the direction of the initial anchor in theory sending an afferent signal through the integumentary system to facilitate an efferent response to facilitate opening of the eyelid. The tension on the Tape also acted to mechanically lift the lid.

**Supplement 3: Device Application Training Detailed Methods**

MLP Training on Self Application: Participants were seated in front of a mirror and provided with an applicator (Figure 2D) dipped in blue eye shadow and asked to try to make a mark with the eye shadow in the center of the eyelid, as though applying the eyelid magnet. This was repeated with feedback 2-3 times, more as needed, before attempting to apply the actual lid magnet. Training in self-application was repeated until the clinician and participant were satisfied it could be done independently at home by the participant or a caregiver. Study staff discontinued the visit and scheduled a follow-up if they could not become proficient after a few attempts, particularly if the lid was becoming red and irritated. Fortunately, this was never an issue for any participant. A follow up video call was conducted a few days later to assure the self-application was going well, or to re-instruct. Paper instructions were sent with the participant along with supplies for the week.

KT Tape Training on Self Application: Participants were trained to apply the tape as described above. They were seated in front of a mirror and provided the Tape swatches with backing still attached. Participants we directed to find a landmark on their forehead as the starting anchor point and adjust from there with repeated attempts as needed to open the eye while allowing complete volitional blink. Training in self-application was performed and repeated until the clinician and participant were satisfied it could be done independently at home by the participant or a caregiver. Staff were instructed to discontinue the visit and schedule a follow-up if the participant could not become proficient before the lid became red and irritated. Fortunately, this was never an issue for any participant. A follow up video call was conducted a few days later to assure the self-application was going well or to re-instruct. The participant left with printed instructions and a Tape supply kit that included the 6 various Tape sizes they could experiment with to self-evaluate performance at home during the practice week. During the study week, only the preferred size, determined during the practice week, was used.

**Supplement 4: Details on Blinding and Code Breaking**

*Participant Masking:* It was explained that 2 different non-surgical treatments were being evaluated. When the sham MLP was placed on the participant at timepoint T_2_, and it was explained that a slightly different configuration was being tested. Participants were not informed about the sham comparison or the active control (Tape) until after the crossover was completed and they had selected a preferred treatment.

*Statistical Analyst Masking*

The scientist performing data analysis (SP) was independent, masked to treatment assignment, and was only involved in the analysis, writing, and interpretation of results. The statistical analyst was unmasked only after the analyses were completed and a formal analysis report had already been generated.

*Code Breaker*

The code was kept in a password-protected file created by a study technician. The file was never accessed by the PI, Co-I, statistical analyst, or other study staff. A study technician broke the code after completion of the final data analyses.

*Data Processor*

The data processor (PS) who conducted manual measurements of the eyelid IPF data using ImageJ could not be fully masked because it was not possible to fully obscure the view of the devices in the video frames being measured. To minimize bias, a processor was selected who did not work for the principal or co-investigator (KH & EP). The data processor had no financial interest in the MLP or Tape.

*Management of potential conflict of interest*

Both the PI and one of the Co-Is (KH & EP) are named on a patent for the MLP device, and so have financial interest which was reviewed by the institutional office of compliance resulting in a management plan. This included the use of double-blind RCT methodology, an independent scientist for data analysis, and an independent safety monitor. The Co-I (EP), was involved only in engineering and results interpretation.

**Supplement 5: Separate attachment**

**Supplement 6: Patient Reported Outcome Plots**

| A.  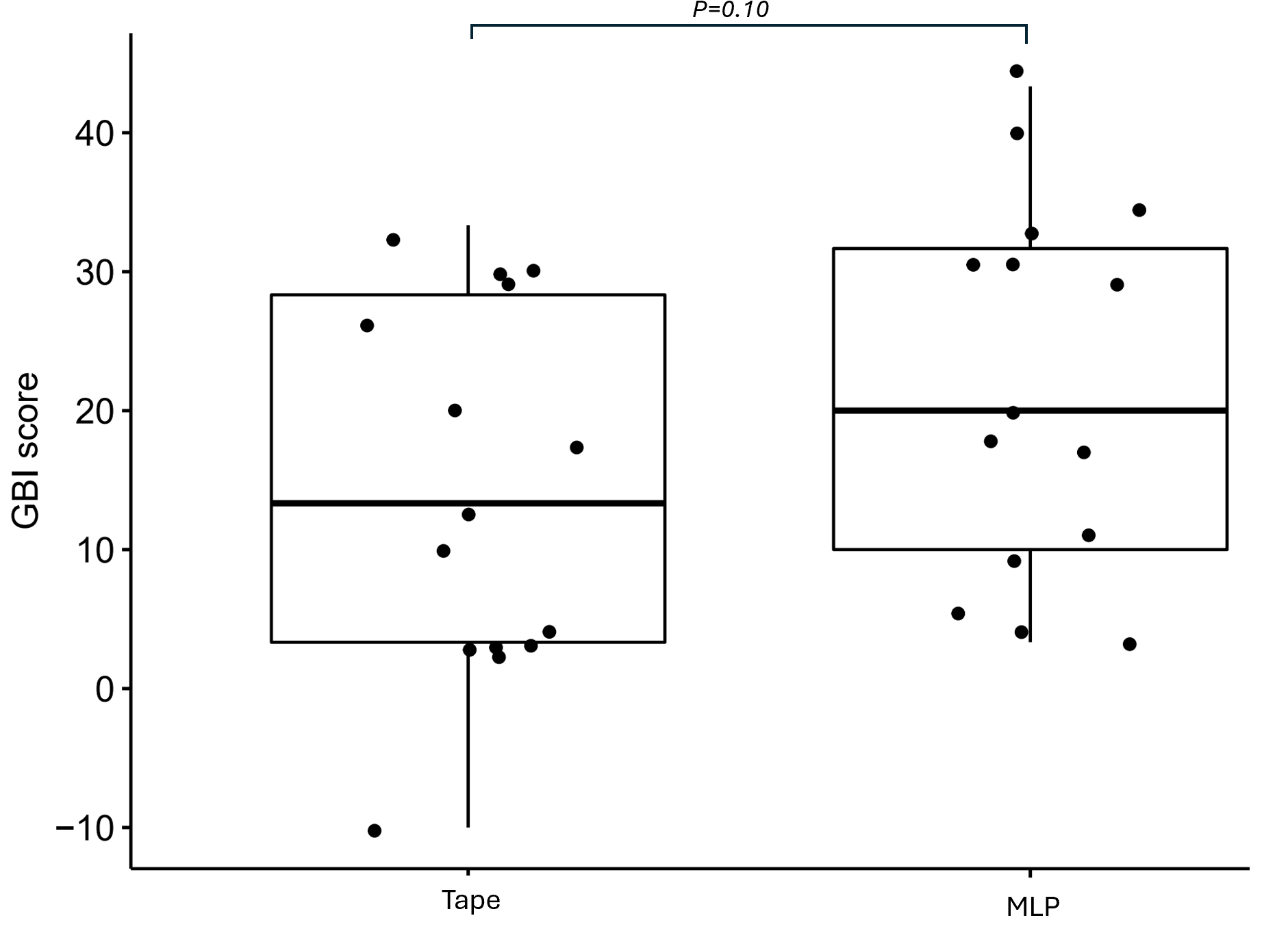 | B.  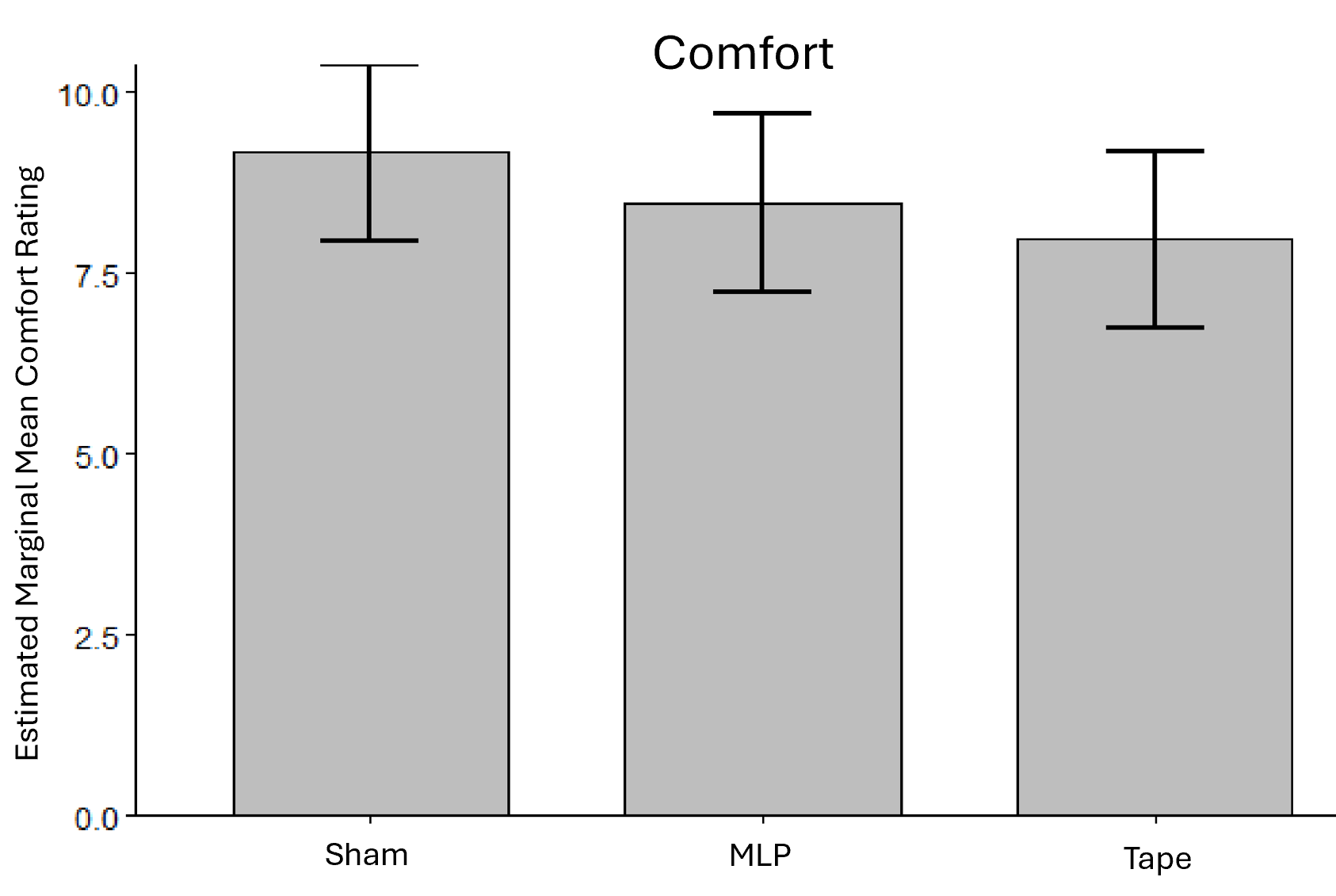 |
| --- | --- |
| Figure 4. Patient reported outcomes for A) the GBI significant perceived benefit of both Tape and MLP (>0) with a marginally more benefit of the MLP, and B) good comfort overall for both devices with no significant difference. | |
